# Numerical simulation study on multifield coupling of enhanced geothermal systems under different fracture characteristics

**DOI:** 10.1371/journal.pone.0319376

**Published:** 2025-06-04

**Authors:** Hanbo Cui, Xintong Jiang, Zongyun Mo, Shenghao Guo, Changshuang Zhao, Weitan Zhuang, Fei Guo

**Affiliations:** 1 School of Architecture and Civil Engineering, Anhui Polytechnic University, Wuhu, China; 2 Engineering Research Center of Anhui Green Building and Digital Construction, Anhui Polytechnic University, Wuhu, China; 3 School of Materials Science and Engineering, Chang’an University, Xi’an, China; China University of Mining and Technology, CHINA

## Abstract

Fractures are key geological features in hot dry rock structures and fulfill a decisive role in determining productivity and reservoir stability. Adopting the Xudong fault zone in the Songliao Basin as the research object, a multifracture heat extraction model was constructed using COMSOL software to systematically analyze productivity and various field under different numbers and locations of horizontal and vertical fractures. Moreover, the influences of vertical fracture connectivity and the characteristics of seepage and heat transfer between the upper and lower rock layers on the temperature field were evaluated. The findings are as follows: (1) The production flow obtained with nine horizontal fractures is 2.25 to 2.28 times that obtained with four horizontal fractures. Increasing the number of horizontal fractures also increases the production temperature and heat extraction efficiency at the early stages of heat extraction but reduces productivity at the later stages and adversely affects reservoir stability. After 30 years of heat extraction, the production temperature, average subsidence, maximum subsidence, and average in situ stress obtained with nine horizontal fractures are 79.38% and 1.87, 1.61, and 1.45 times, respectively, those obtained with four horizontal fractures. (2) The influence of the number of vertical fractures on the geothermal reservoir characteristics is similar to but slightly smaller than that of horizontal fractures. However, the influences of vertical fractures on the production flow at the early stages and the maximum reservoir temperature at the later stages are opposite to those of horizontal fractures. When vertical fractures are located close to the injection well, productivity is low at the early stages but high at the later stages. The maximum subsidence, average in situ stress, and maximum in situ stress slightly increase, whereas the average subsidence decreases. (3) After 30 years of heat extraction, the average reservoir temperature is highest when seepage and heat transfer between the upper and lower rock layers occur and when vertical fractures do not penetrate the reservoir. When these conditions are reversed, the average temperature is lowest, with the former approximately 0.42°C higher than the latter. The findings of this study provide a reference for the construction of reservoir fracture systems.

## Introduction

Enhanced geothermal systems (EGSs) are fracture systems with favorable connectivity installed in deep tight hot dry rock (HDR) reservoirs via artificial fracturing. The key to stable EGS operation lies in whether the modified fracture system provides sufficient stability to withstand in situ stress and temperature changes, thus ensuring continuous and efficient extraction of thermal energy. Therefore, the study of the influences of fracture characteristics on productivity and various fields during EGS heat extraction has important engineering significance.

Scholars have extensively investigated the relationship between the internal fracture characteristics of reservoirs and heat extraction. Some experts have explored the influence of fracture density and surface area on EGS heat extraction. Shook et al. [1] applied the tracer test method and determined that the fracture density and surface area within a given rock mass after artificial fracturing determine the final production temperature and power generation. Zhou et al. [2] noted that there exists a positive correlation between the heat extraction efficiency and fracture density. Sanyal et al. [3] demonstrated that this relationship varies; notably, when the volume proportion of the fractured zone exceeds 40%, the correlation between the heat extraction efficiency and fracture density significantly decreases. Other experts have studied the relationship between the fracture width and heat extraction. Gao [4], Liao et al. [5], and Yang et al. [6] proposed that a decrease in the reservoir temperature induces thermal stress, which causes rock mass shrinkage and fracturing; a larger temperature difference results in wider fractures, which in turn lead to a decrease in the injection pressure, thus enhancing the production flow and facilitating efficient thermal energy extraction. Tang et al. [7] explored this phenomenon from a production temperature perspective, observing that although an increase in the fracture width enhances the thermal energy extraction efficiency, the resulting production temperature decreases. Li et al. [8] presented a different perspective, suggesting that a greater fracture width could cause a short-circuit of the working fluid, in turn negatively affecting the final thermal energy extraction efficiency. J. Zhang et al. [9] reported that the fracture width is not only related to productivity but also affects temperature decrease zone of the reservoir. The fracture length is another important indicator considered by experts and scholars. B. Zhang et al. [10] noted that the properties of the injection working fluid affect the propagation length and path of fractures and determine the variation in the production temperature. Shi et al. [11] demonstrated that longer fractures are conducive to high-quality extraction of thermal energy. From the perspective of reservoir temperature decrease, Ekneligoda et al. [12], Sun et al. [13], and Ma et al. [14] determined that the most suitable fracture length is 600 m, and they provided relevant indicators for the fracture length most conducive to efficient thermal energy extraction. In addition, the influence of the fracture infiltration capacity on heat extraction cannot be ignored. Mclean et al. [15] emphasized that during heat extraction, reservoir fractures must exhibit sufficient permeability to meet the production demand of the working fluid. Abe et al. [16] explored this topic in depth and reported that a complex fracture system exhibits high permeability, which helps increase the heat extraction efficiency. Kelvin et al. [17] reported that the in situ stress is a key indicator that affects the reservoir permeability and determines the timing of thermal breakthrough, and an appropriate in situ stress can ensure working fluid flow uniformity. Other experts have investigated the influence of fracture characteristics on the EGS heat extraction process in terms of the fracture roughness [18–20] and fracture uniformity [21–22].

On the basis of recent studies, most researchers have employed the production temperature and heat extraction efficiency as criteria to assess the feasibility of long-term reservoir development. However, studies accounting for the influence of reservoir stability on the extraction lifespan are relatively rare, and the few explorations available are neither systematic nor comprehensive. Notably, the degree of reservoir subsidence and in situ stress changes also determine whether a geothermal system can maintain stable and high-quality operation and production. Most studies on fracture characteristics have focused on the influence of factors such as the fracture length, width, and permeability on the heat extraction process. Research on the construction of fracture models has focused mainly on horizontal fractures, whereas studies on the relationship between horizontal and vertical fractures are rare. How to effectively use naturally occurring vertical fractures is a key factor for realizing efficient EGS extraction. In addition, previous studies on the reservoir temperature have typically focused on the production temperature or heat extraction efficiency. Changes in the average reservoir temperature and the maximum reservoir temperature are also important indicators in determining the ultimate productivity and ensuring sustainable and stable reservoir extraction conditions.

In this study, an EGS multifracture heat extraction model was established on the basis of geothermal geological data from the Xujiaweizi Xudong fault zone in the Songliao Basin combined with numerical simulation technology using COMSOL software. This model aims to analyze variations in reservoir productivity (production temperature, production flow, and heat extraction efficiency), temperature fields (target area temperature field, average reservoir temperature, and maximum reservoir temperature), displacement fields (target area displacement field, average reservoir subsidence, and maximum reservoir subsidence), and stress fields (target area stress field, average reservoir stress, and maximum reservoir stress) under different conditions such as the number and locations of horizontal and vertical fractures. Moreover, the mechanisms underlying the influences of the connectivity characteristics of vertical fractures and the characteristics of heat transfer and seepage between the upper and lower rock layers on the temperature field were examined in detail. This study provides reference data for the construction of suitable reservoir fracture systems to ensure the continuous and stable development of HDR resources.

## Methods

**Fig 1:**
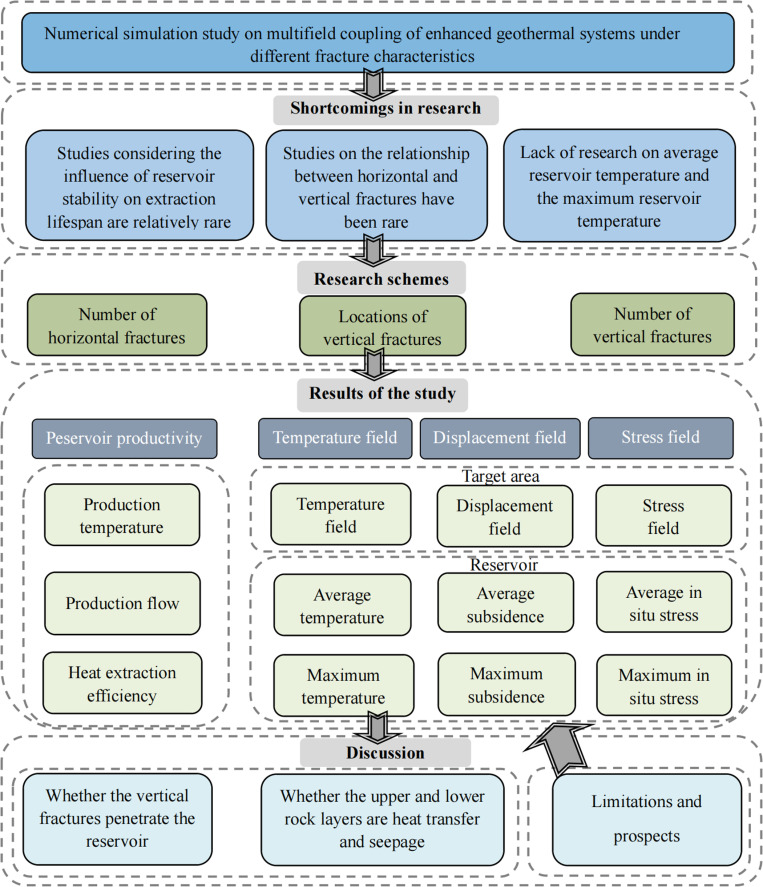
Technical route.

The technical route is shown in Fig 1.

### Geological background

**Fig 2:**
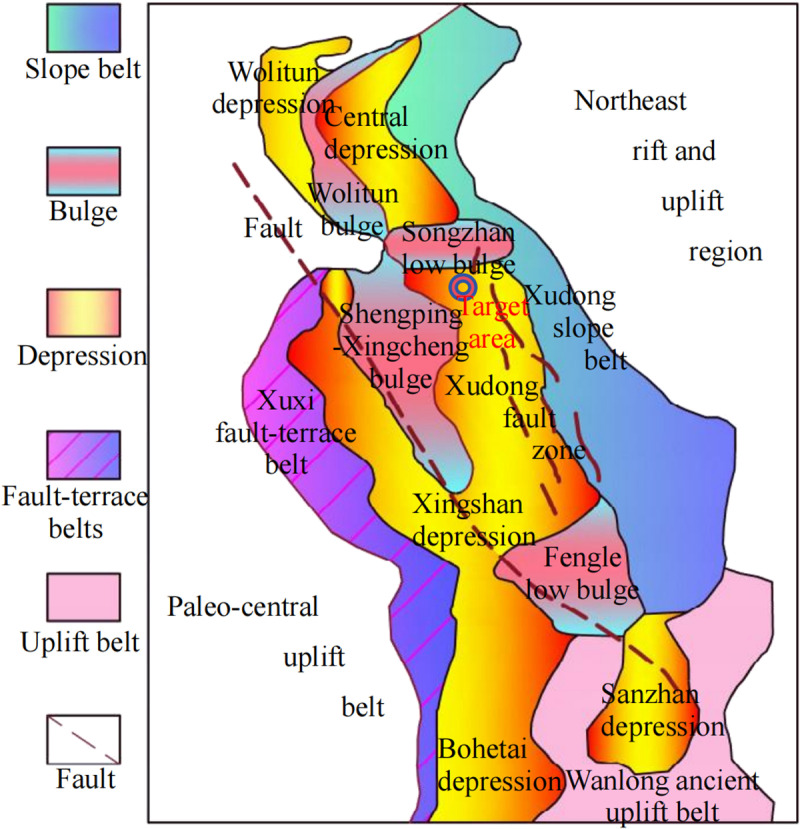
HDR mining target area. Different colored fillings represent different geological features, and the research object is located in the Xudong Fault Zone.

The Xudong fault zone is located between the paleo-central uplift belt and the northeastern rift and uplift region, and it is an important secondary tectonic unit within the Xujiaweizi fault depression. High-precision aeromagnetic survey data indicate that the geological background of the Xudong fault zone is complex. Notably, it has experienced multiple tectonic movements, resulting in significant fault zones, and numerous longitudinal faults occur. The strata within the Xudong fault zone exhibit significant sedimentary thicknesses, providing a suitable caprock for thermal energy storage. The deep strata in the Xudong fault zone contain large quantities of magmatic rocks, with high terrestrial heat flow levels and multiple geothermal resource anomaly points, where the temperature at the same depth is 8–10°C higher than that of the surrounding strata. High-temperature rock masses (100–206°C) have been drilled at various locations such as in wells Xushen-1, Xushen-4, Xushen-22, and Xushen-33 [23–27]. In addition, the Xudong area contains abundant water resources, with the Nenjiang River, Songhua River, and Liaohe water systems flowing through the area, providing abundant working fluids for geothermal resource extraction. In summary, the Xujiaweizi Xudong fault zone meets the prerequisites for consideration as a target area for heat extraction. The HDR mining target area is shown in Fig 2 (modified from Wang [27]).

### Model establishment

**Fig 3:**
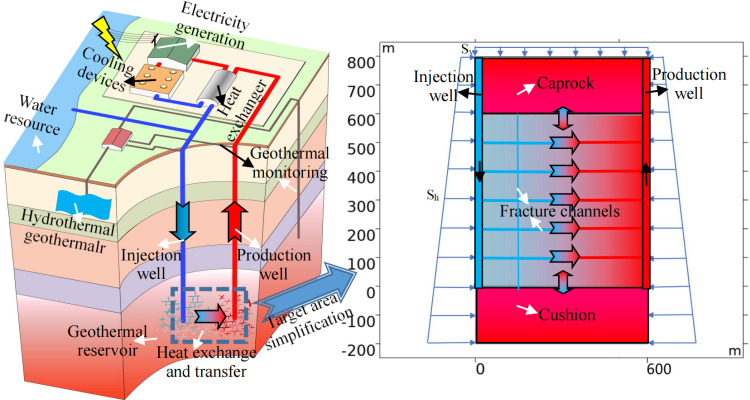
HDR thermal mining model and simplified model.

To facilitate analysis, the reservoir was simplified and modeled according to the HDR mining conditions and the actual target area. The model encompasses an injection well (on the left side of the model), a production well (on the right side of the model), fracture channels (parallel or perpendicular to the reservoir, which simulate horizontal fracture channels or vertical natural fractures, respectively), a matrix rock mass, and upper and lower rock layers (caprock and cushion, respectively). Water is injected into the geothermal reservoir via the injection well, with most water flowing into the fractures and a small portion entering the matrix rock mass. After the completion of heat exchange and transfer between the rock mass and fluid, water flows out from the production well via the fractures or matrix for utilization. Part of the fluid flows into the upper and lower rock layers via vertical fractures and the matrix, resulting in the exchange and loss of fluid and heat. The HDR thermal mining model and the simplified model are shown in Fig 3 (modified from Yu [23]).

Focusing on seepage and heat transfer processes in the reservoir, nonessential factors influencing heat extraction were ignored, and the following model and heat extraction process assumptions were made:

(1) Microfractures are ignored, and only the main fracture channels are considered; artificial fractures are simplified as horizontal fracture channels, whereas natural fractures are simplified as vertical fracture channels;
(2) Water phase changes during seepage and heat transfer are ignored, and water is assumed to always remain in the liquid state;
(3) Chemical reactions between water and rock mass are ignored [28];
(4) Changes in the geothermal gradient are ignored;
(5) Differences in rock types and rock anisotropy are ignored, and the reservoir is assumed to comprise homogeneous rock with the same properties.

### Model parameters

In situ stresses were obtained via the horizontal and vertical in situ stress equations proposed by Valley [29]:


$$S_{h}=-1.17+22.95*z$$ (1)

$$S_{v}=-1.3+25.5*z$$ (2)

where $S_{h}$ is the horizontal in situ stress, MPa; $S_{v}$ is the vertical in situ stress, MPa; and *z* is the extraction depth, km.

Based on geological and geothermal survey data from the Xudong fault zone [23–27] as well as the thermodynamic indicators of granite and water, the model parameters are listed in Table 1.

**Table 1:** Model parameters.

	Model parameter	Value		Model parameter	Value
**Matrix rock mass**	Temperature (∘C)	206	**Fracture**	Pore pressure (MPa)	33.345
	Depth (km)	3800–4400		Permeability ($m^{2}$)	$1\times 10^{-10}$
	Thermal expansivity ($\circ C^{-1}$)	$5\times 10^{-6}$		Specific storage ($Pa^{-1}$)	$1\times 10^{-9}$
	Porosity (%)	4	**Cushion**	Thickness (m)	200
	Poisson’s ratio	0.15	**Caprock**	Thickness (m)	200
	Specific storage ($Pa^{-1}$)	$1\times 10^{-8}$	**Well**	Well spacing (m)	600
	Permeability ($m^{2}$)	$1.0\times 10^{-18}$	**Water**	Density ($kg/m^{3}$)	1005
	Thermal conductivity ($W/m.^{\circ }C$)	2.86		Specific heat capacity ($J/kg.^{\circ }C$)	3993
	Specific heat capacity ($J/kg.^{\circ }C$)	971		Injection temperature (∘C)	40
				Thermal conductivity ($W/m.^{\circ }C$)	0.644
	Density ($kg/m^{3}$)	3300		Dynamic viscosity (Pa.s)	$6.56\times 10^{-4}$

### Governing equations

**Fig 4:**
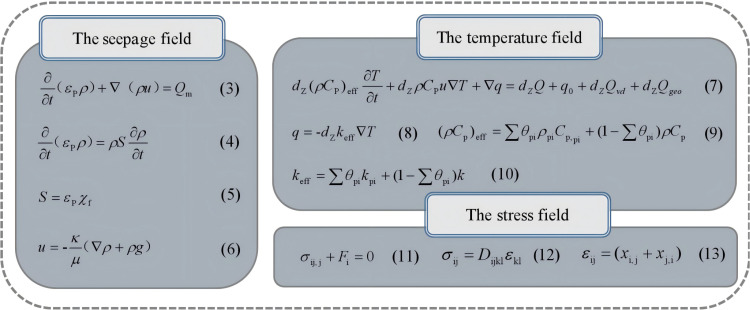
Governing equations for each field.

Based on thermo–hydro–mechanical coupling theory, an EGS multifracture heat extraction model was established to describe the variations in the seepage, temperature, and mechanical fields. The governing equations for each field are shown in Fig 4, and the meanings of the parameters in the governing equations are provided in Table 2.

**Table 2:** Meanings of the parameters in the governing equations.

Model parameter	Meaning	Model parameter	Meaning	Model parameter	Meaning
*t* (a)	Time	$d_{Z}$ (m)	Thickness	$\theta_{pi}$	Volume fraction of the porous matrix
$\varepsilon_{P}$ (%)	Rock porosity	$C_{p}$ ($J/kg.^{\circ }C$)	Specific heat capacity of water	$\rho_{pi}$ (kg/m^3^)	Rock density
ρ (kg/m^3^)	Water density	eff	Equivalent physical property	$k_{pi}$ ($W/m.^{\circ }C$)	Thermal conductivity of rock
u	Velocity field	*T* (°C)	Temperature	${C_{p,}}_{pi}$ ($J/kg.^{\circ }C$)	Specific heat capacity of rock
$Q_{m}$ ($kg/(m^{3}s)$)	Mass source term	*Q* (kg/s)	Heat flux	$\sigma_{ij,j}$ (MPa)	Second-order stress tensor
*S* (1/Pa)	Storativity	*Q*	Heat source	$F_{i}$ (kN)	Body force
$\chi_{f}$	Compressibility	$q_{0}$ (kg/s)	Initial heat flux	$D_{ijkl}$	Strain‒displacement tensor
κ (m^2^)	Rock permeability	$Q_{vd}$	Viscous dissipation heat source	$\varepsilon_{ij}$	Strain tensor
μ (Pa.s)	Hydrodynamic viscosity	$Q_{geo}$	Geothermal heat source	$x_{i,j}$	Displacement tensor
*g* (m/s^2^)	Gravity vector	*k* ($W/m.^{\circ }C$)	Thermal conductivity of water	$x_{j,i}$	Displacement tensor

### Research schemes

With reference to the current engineering needs and research overview, 16 simulation schemes were designed to investigate the influence of fracture characteristics on the HDR heat extraction process:

(1) Number of horizontal fractures (considering that reservoir thermal energy is far from fully utilized when the number of horizontal fractures ranges from 1 to 3): A total of 4, 5, 6, 7, 8 and 9 horizontal fracture channels were evenly arranged throughout the reservoir;
(2) Location of the vertical fractures (in the presence of five horizontal fractures, the reservoir exhibits high thermal energy utilization efficiency, and the influence between fracture channels is limited; therefore, the number of horizontal fractures was set to five): One vertical fracture was arranged at distances of 100, 200, 300, 400, or 500 m from the production well;
(3) Number of vertical fractures (the number of horizontal fractures is five): A total of 1, 2, 3, 4, and 5 vertical fractures were evenly arranged in the reservoir.

### Grid verification

**Fig 5:**
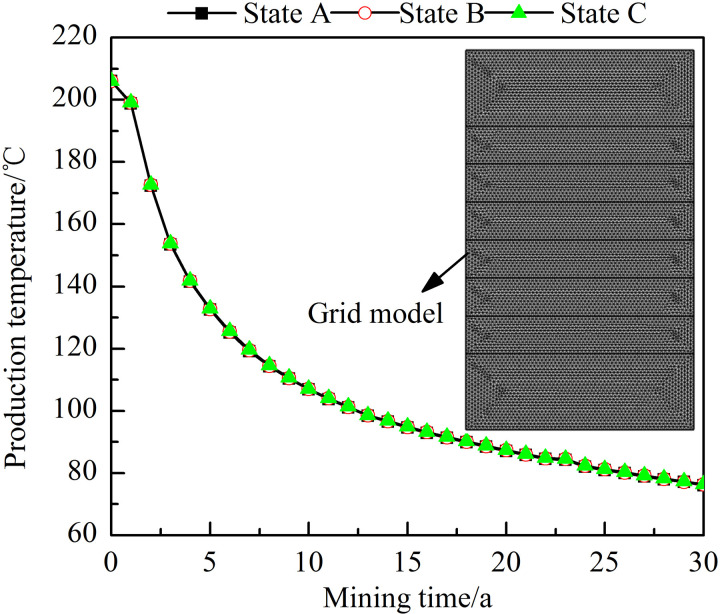
Grid independence verification results. The dots represent the production temperature at different times (year). The number and density of grids in states A, B, and C gradually increase.

The grid number and density affect the final calculation results, making it necessary to perform grid independence verification of the models. Since the number of grids varies between the models, it would be computationally costly to verify all models. Therefore, the production temperature of the model with five horizontal fractures was selected for verification. The simulated heat extraction period is 30 years, with a step size of 0.01 year. The grid independence verification scheme is detailed in Table 3, and the verification results are shown in Fig 5. The study results indicate that when the grid number increases from fine state A to extremely fine state C, the difference in the production temperature variation is very small but nonnegligible. Therefore, to ensure the calculation accuracy, the extremely fine state C was selected for all models.

**Table 3:** Grid independence verification scheme.

Groups	Grid encryption	Statistical data				
		Mesh vertices	Triangular elements	Edge elements	Vertex units	Number of units
**State A**	Fine	764	1432	264	24	1432
**State B**	Ultra fine	2011	3848	472	24	3848
**State C**	Extremely fine	8078	15828	926	24	15828

### Simulation results and analysis

#### Influence of fracture characteristics on the temperature field.

In view of the similarity of the trends obtained after varying the simulation conditions and the limited space available, only the temperature, displacement, and stress fields under select working conditions of the number of horizontal fractures (4 and 9), the location of the vertical fractures (100 and 500 m), and the number of vertical fractures (1 and 5) are described in depth.

**Fig 6:**
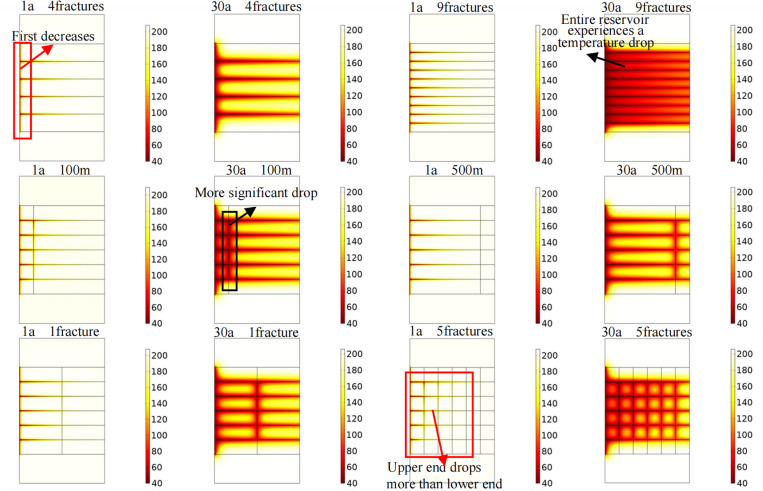
Influences of different fracture characteristics on the temperature field in the target area. 1a and 30a represent different heat extraction times. The figure in the first row shows the variation pattern of the temperature field in the target area when the number of horizontal fractures is 4 and 9. The figure in the second row shows the variation of temperature field in the target area when the vertical fracture is 100m and 500m away from the injection well. The figure in the third row shows the variation pattern of the temperature field in the target area when the number of vertical fractures is 1 and 5.

Fig 6 shows the influences of different fracture characteristics on the temperature field in the target area. At the early stages of extraction, the temperature first decreases in the junction area of the injection well and horizontal fractures. With fluid injection, the temperature decrease area expands from the injection well to the production well and from the reservoir to the upper and lower rock layers. Late stage of heat extraction, almost the entire reservoir exhibits varying degrees of temperature decrease, and the temperature in the upper and lower rock layers changes. With increasing number of horizontal fractures, the temperature decrease area increases significantly. After 30 years of heat extraction, when four horizontal fractures are implemented, there are still areas with high temperatures between the horizontal fractures. When nine horizontal fractures are implemented, almost the entire reservoir exhibits a significant decrease in the temperature. Therefore, appropriately increasing the number of horizontal fractures can effectively increase the rate of thermal energy extraction from the reservoir. When the vertical fracture is close to the injection well, the degree of cooling near the fracture is more pronounced. When the number of vertical fractures in the reservoir is increased to 5, after 1 year of heat extraction, the temperature decrease near the fractures at the upper end of the reservoir is greater than that in the surrounding areas of the fractures at the lower end of the reservoir. After 30 years of heat extraction, the temperature decrease area is significantly larger than that when implementing only one fracture.

**Fig 7:**
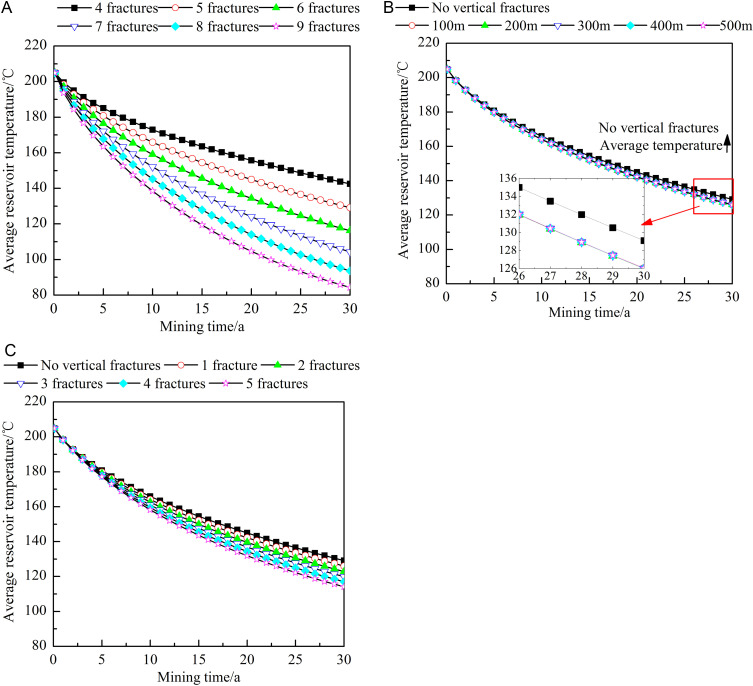
Influences of different fracture characteristics on the average reservoir temperature. A) Number of horizontal fractures. B) Locations of the vertical fractures. C) Number of vertical fractures. The dots in the figure represent the average reservoir temperature at different heat extraction times (0-30 year). The figure (a) shows the average reservoir temperature when the number of horizontal fractures is 4-9. The figure (b) shows the average reservoir temperature when no vertical fractures or the vertical fracture is 100-500m away from the injection well. The figure (c) shows the average reservoir temperature when no vertical fractures or the number of vertical fractures is 1–5.

Fig 7 shows the influence of different fracture characteristics on the average reservoir temperature. As the heat extraction process progresses, the average reservoir temperature under each heat extraction condition decreases. As shown in Fig 7(a), the larger the number of horizontal fractures is, the greater the decrease in the mean reservoir temperature. After 30 years of heat extraction, the average reservoir temperature obtained with nine horizontal fractures is approximately 59.13% of that obtained with four horizontal fractures. Fig 7(b) shows that the reservoir temperature decrease is larger when there are vertical fractures than when there are no vertical fractures. The change in the location of the vertical fractures exerts an extremely limited impact on the average reservoir temperature and can be neglected. As shown in Fig 7(c), the more vertical fractures there are, the lower the average reservoir temperature. After 30 years of heat extraction, the average reservoir temperature obtained with five vertical fractures is approximately 90.56% of that obtained with one vertical fracture.

**Fig 8:**
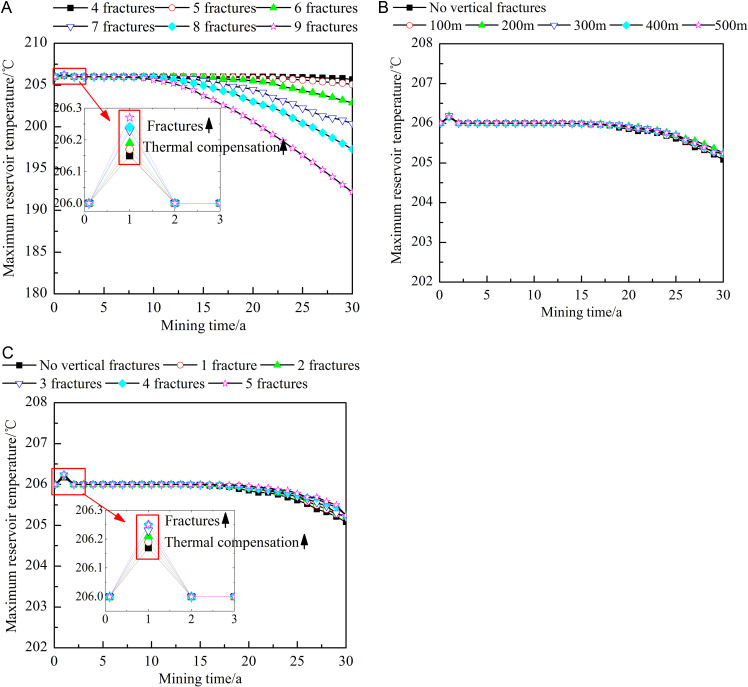
Influences of different fracture characteristics on the maximum reservoir temperature. A) Number of horizontal fractures. B) Locations of the vertical fractures. C) Number of vertical fractures. The dots in the figure represent the maximum reservoir temperature at different heat extraction times (0-30 year). The figure (a) shows the maximum reservoir temperature when the number of horizontal fractures is 4-9. The figure (b) shows the maximum reservoir temperature when no vertical fractures or the vertical fracture is 100-500m away from the injection well. The figure (c) shows the maximum reservoir temperature when no vertical fractures or the number of vertical fractures is 1-5.

Fig 8 shows the influence of different fracture characteristics on the maximum reservoir temperature. As shown in Figs 6 and 8, at the early stages of heat extraction, the maximum temperature near the junction area between the injection well and the upper and lower rock layers is slightly higher than the initial temperature of 206°C. This is due to the large temperature difference generated between the reservoir and the upper and lower rock layers during heat extraction, causing heat transfer from the upper and lower rock layers to the reservoir, which results in a thermal compensation effect. As the heat extraction process progresses, the maximum reservoir temperature decreases to varying degrees under the different fracture parameters, and the location of the maximum temperature constantly shifts toward the production well. As shown in Fig 8(a), the larger the number of horizontal fractures is, the greater the thermal compensation effect and the higher the maximum reservoir temperature at the initial stages of heat extraction, but this is accompanied by earlier thermal breakthrough and a larger overall temperature decrease. After 30 years of extraction, the maximum reservoir temperature obtained with 9 horizontal fractures is 93.45% of that obtained with 4 horizontal fractures. Fig 8(b) shows that the location of the vertical fractures slightly influences the maximum reservoir temperature. Therefore, the influence of this factor is not assessed further. Fig 8(c) shows that with increasing number of vertical fractures, heat flow from the upper and lower rock layers to the reservoir increases, resulting in a significant thermal compensation effect. Therefore, the maximum reservoir temperature increases at the early stages of heat extraction. Compared with the influence of the number of horizontal fractures, the occurrence of thermal breakthrough is slightly delayed when more vertical fractures are implemented. Moreover, the maximum reservoir temperature at the middle and late stages of heat extraction is positively correlated with the number of vertical fractures. Thus, it can be inferred that the presence of vertical fractures leads to a greater thermal compensation effect in the reservoir.

#### Influence of fracture characteristics on the production temperature.

**Fig 9:**
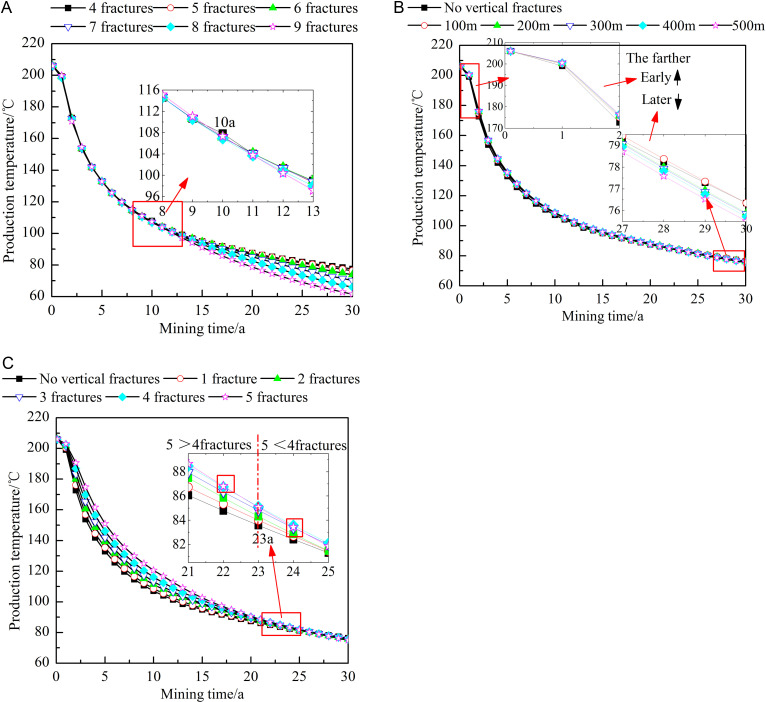
Influences of different fracture characteristics on the production temperature. A) Number of horizontal fractures. B) Locations of the vertical fractures. C) Number of vertical fractures. The dots in the figure represent the production temperature at different heat extraction times (0-30 year). The figure (a) shows the production temperature when the number of horizontal fractures is 4-9. The figure (b) shows the production temperature when no vertical fractures or the vertical fracture is 100-500m away from the injection well. The figure (c) shows the production temperature when no vertical fractures or the number of vertical fractures is 1-5.

Fig 9 shows the influence of different fracture characteristics on the production temperature. After fluid injection, the temperature between the matrix and water quickly reaches equilibrium. At the early stages of heat extraction, the production temperature under all fracture characteristics remains 206°C, which is the initial reservoir temperature. As the process of heat extraction progresses, the production temperature under all fracture characteristics shows a decreasing trend, with a high decrease rate at the early stages and a relatively low decrease rate at the middle and late stages of heat extraction. As shown in Fig 9(a), at the early stages of heat extraction, increasing the number of horizontal fractures slightly increases the production temperature. This occurs because an increase in the number of horizontal fractures increases the contact area between the fractures and the matrix, thus causing more heat to flow out. After heat extraction for around 10 years, this trend changes. Notably, with increase in the number of horizontal fractures, the production temperature decreases instead. This occurs because there is significant heat loss at this time, namely, the more horizontal fractures there are, the higher the heat loss. Thermal energy cannot be replenished in time, resulting in a lower production temperature. After 30 years of heat extraction, the production temperature obtained with nine horizontal fractures is approximately 79.38% of that obtained with four horizontal fractures. As shown in Fig 9(b), the farther the vertical fractures are from the injection well, the higher the production temperature at the early stages and the lower the production temperature at the later stages. Upon investigation, it is found that at the early stages of extraction, heat is first consumed near the injection well and the horizontal fracture channels. Owing to the dense nature of the reservoir rock, the rock farther from the injection well and horizontal fracture channels maintains high thermal energy. Therefore, adding vertical fractures far from the injection well results in a higher production temperature at the early stages of heat extraction, but this increases the overall heat consumption during extraction, yielding a lower production temperature at the later stages. As shown in Fig 9(c), during the first 23 years of heat extraction, the larger the number of vertical fractures is, the higher the production temperature. However, thereafter, this trend gradually changes, and the production temperature decreases with increasing number of fractures. Notably, the influences of increasing the number of vertical or horizontal fractures on the production temperature are similar. Since the influence of the former is less than that of the latter, the above phenomenon emerges slightly later in the former.

### Influence of fracture characteristics on the production flow

**Fig 10:**
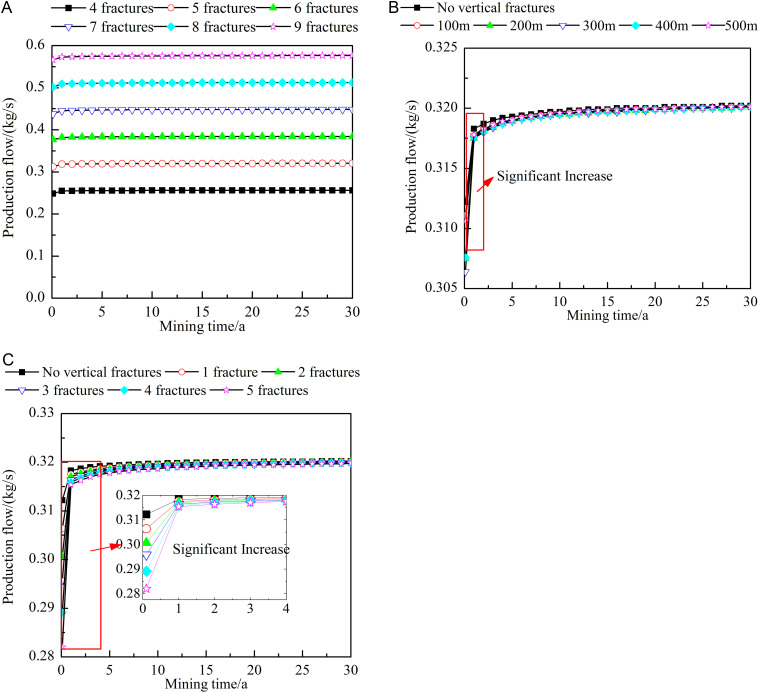
Influences of different fracture characteristics on the production flow. A) Number of horizontal fractures. B) Locations of the vertical fractures. C) Number of vertical fractures. The dots in the figure represent the production flow at different heat extraction times (0-30 year). The figure (a) shows the production flow when the number of horizontal fractures is 4-9. The figure (b) shows the production flow when no vertical fractures or the vertical fracture is 100-500m away from the injection well. The figure (c) shows the production flow when no vertical fractures or the number of vertical fractures is 1-5.

Fracture characteristics directly determine the flow path of the working fluid. Therefore, it is important to study the influence of fracture characteristics on the production flow. Fig 10 shows the influence of different fracture characteristics on the production flow. As the process of heat extraction progresses, the production flow increases under the different fracture characteristics, with a clear increase at the early stages and gradual stabilization at the later stages. As shown in Fig 10(a), when the number of horizontal fractures is increased, the production flow increases approximately proportionally. After 0.1 year of heat extraction, the production flow obtained with nine horizontal fractures is approximately 2.28 times that obtained with four horizontal fractures. After 30 years of heat extraction, the former is approximately 2.25 times the latter. Therefore, increasing the number of horizontal fractures within a reasonable range is equivalent to increasing the number of connecting channels between the injection and production wells, which promotes the production flow. Fig 10(b) and Fig 10(c) show that the influence of the number and location of the vertical fractures on the production flow is greater at the early stages than at the later stages of heat extraction. At the early stages of heat extraction, when vertical fractures are present in the reservoir, the production flow decreases, and the decrease is relatively maximum when the vertical fractures are located 200–300 m from the injection well. An increase in the number of vertical fractures results in lower production flow. This occurs because a portion of the injected fluid flows towards the production well, while another portion is injected into vertical fractures and matrix. The amount of working fluid injected into vertical fractures is greater than that into matrix. Therefore, when there are vertical fractures in the reservoir or the number of vertical fractures increases, the production flow rate will decrease accordingly. As the vertical fractures are increasingly filled with the working fluid, the difference in the production flow among the various working conditions gradually decreases.

#### Influence of fracture characteristics on the heat extraction efficiency.

The heat extraction efficiency can be obtained with the equation proposed by Chen [30]:


$$W=Q_{pro}C_{p}T_{pro}$$ (14)

where W is the heat extraction efficiency, MW; and $T_{pro}$ is the production temperature,°C.

**Fig 11:**
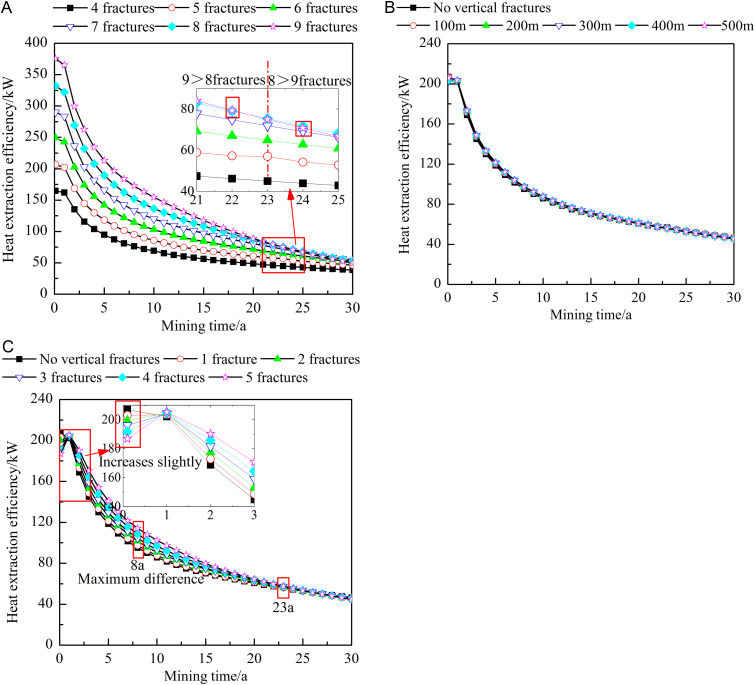
Influences of different fracture characteristics on the heat extraction efficiency. A) Number of horizontal fractures. B) Locations of the vertical fractures. C) Number of vertical fractures. The dots in the figure represent the heat extraction efficiency at different heat extraction times (0-30 year). The figure (a) shows the heat extraction efficiency when the number of horizontal fractures is 4-9. The figure (b) shows the heat extraction efficiency when no vertical fractures or the vertical fracture is 100-500m away from the injection well. The figure (c) shows the heat extraction efficiency when no vertical fractures or the number of vertical fractures is 1-5.

Fig 11 shows the influence of different fracture characteristics on the heat extraction efficiency. As shown in Fig 11(a), at the early stages of heat extraction, the larger the number of horizontal fractures is, the higher the heat extraction efficiency. After 0.1 year of heat extraction, the heat extraction efficiency obtained with 9 horizontal fractures is approximately 2.28 times that obtained with 4 horizontal fractures. At the middle and late stages of heat extraction, the implementation of fewer horizontal fractures results in a higher heat extraction efficiency. After 23 years of extraction, the heat extraction efficiency obtained with 8 horizontal fractures is greater than that obtained with 9 horizontal fractures. This occurs because the heat extraction efficiency is affected by both the production temperature and the production flow. At the early stages, the production flow exerts a greater influence. Notably, the larger the number of horizontal fractures is, the higher the production flow and the higher the heat extraction efficiency. At the later stages, the production temperature increasingly dominates. At this time, the larger the number of horizontal fractures is, the lower the production temperature and the lower the heat extraction efficiency. Combining Fig 11(b) and 11(c), when the location of the vertical fractures is varied, the change trend of the heat extraction efficiency is generally similar to that of the production temperature. Notably, after 0.1 year of heat extraction, an increase in the number of vertical fractures leads to a decrease in the heat extraction efficiency. This phenomenon can be attributed to the fact that the production temperature remains constant at 206°C during this period, rendering the heat extraction efficiency entirely dependent on changes in the production flow rate. Specifically, an increase in the number of vertical fractures causes a reduction in the production flow rate, thereby lowering the heat extraction efficiency. From 0.1–1 year of heat extraction, the heat extraction efficiency shows an upward trend. This occurs because, at this stage, the production flow rate rapidly increases, whereas the production temperature slightly decreases, with the increase in the flow rate outweighing the decrease in the production temperature. As the process of heat extraction progresses, the production flow rate gradually stabilizes, but the production temperature continues to decline, leading to a decrease in the heat extraction efficiency. At this stage, an increase in the number of vertical fractures positively impacts the heat extraction efficiency, with the impact peaking after 8 years of extraction. Moreover, the heat extraction efficiency obtained with five vertical fractures is approximately 1.17 times that obtained with only one vertical fracture. After 23 years of heat extraction, the heat extraction efficiency decreases with increasing number of vertical fractures. At the middle and later stages of heat extraction, the mechanism by which the number of vertical fractures influences the heat extraction efficiency is similar to that of the number of horizontal fractures, but the impact is slightly smaller.

#### Influence of fracture characteristics on the displacement field.

**Fig 12:**
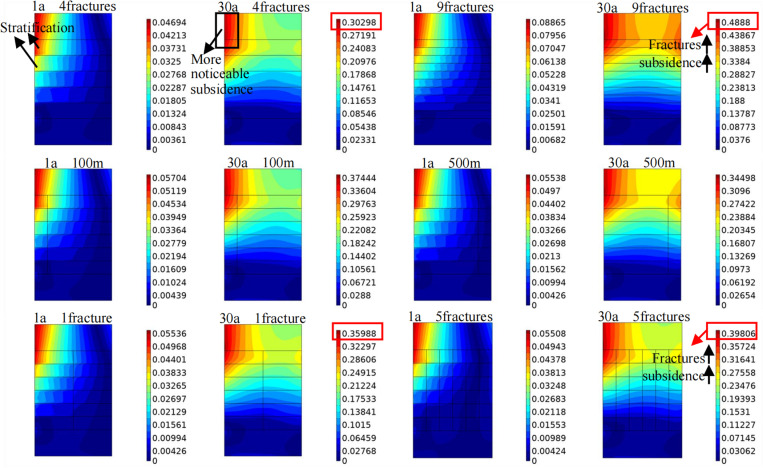
Influences of different fracture characteristics on the displacement field in the target area. 1a and 30a represent different heat extraction times.The figure in the first row shows the variation pattern of the displacement field in the target area when the number of horizontal fractures is 4 and 9. The figure in the second row shows the variation of displacement field in the target area when the vertical fracture is 100m and 500m away from the injection well. The figure in the third row shows the variation pattern of the displacement field in the target area when the number of vertical fractures is 1 and 5.

The displacement field determines the duration of stable extraction of geothermal resources. Thus, in-depth research is urgently needed. Fig 12 shows the influence of different fracture characteristics on the displacement field in the target area. In the early stages of heat extraction, the presence of horizontal fractures causes stratification in reservoir subsidence, i.e., the subsidence at the upper end of the fractures is greater than that at the lower end of the fractures, and the subsidence at the junction between the injection well and each horizontal fracture is greater than that in other areas, especially for the uppermost horizontal fracture. The reason is that this area is affected by heat extraction earlier than other areas, leading to a higher degree of rock shrinkage. As extraction progresses, the reservoir subsides continuously, with more noticeable subsidence closer to the upper part of the target area. In addition, obvious subsidence occurs in the upper region of the reservoir near the production well. A comparison of the displacement field contour plots after 30 years of heat extraction shows that as the number of horizontal or vertical fractures increases, the overall subsidence of the target area increases significantly.

**Fig 13:**
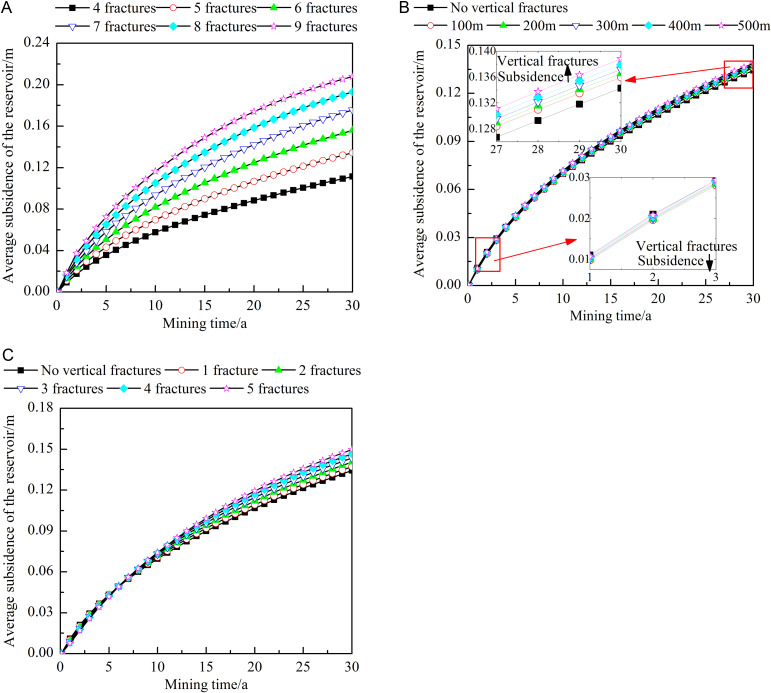
Influences of different fracture characteristics on the average reservoir subsidence. A) Number of horizontal fractures. B) Locations of the vertical fractures. C) Number of vertical fractures. The dots in the figure represent the average reservoir subsidence at different heat extraction times (0-30 year). The figure (a) shows the average reservoir subsidence when the number of horizontal fractures is 4-9. The figure (b) shows the average reservoir subsidence when no vertical fractures or the vertical fracture is 100-500m away from the injection well. The figure (c) shows the average reservoir subsidence when no vertical fractures or the number of vertical fractures is 1-5.

Fig 13 shows the influence of different fracture characteristics on the average subsidence of the reservoir. As shown in Fig 13(a), the more horizontal fractures there are, the greater the average subsidence of the reservoir; as heat extraction progresses, this trend becomes more evident. After 30 years of heat extraction, the average subsidence of the reservoir with 9 horizontal fractures is approximately 1.87 times that with 4 horizontal fractures. As shown in Fig 13(b), in the early stages of heat extraction, the presence of vertical fractures in the reservoir reduces the subsidence of the reservoir. This is because, at the beginning of heat extraction, the working fluid first flows to the vertical fractures, resulting in a lower production flow per unit time, a smaller temperature drop of the reservoir, and a correspondingly smaller overall subsidence. In the later stages of heat extraction, the existence of vertical fractures increases reservoir subsidence, and the larger the distance from the injection well is, the greater the average subsidence of the reservoir. After 30 years of heat extraction, the average subsidence when the vertical fracture is 500 m from the injection well is approximately 1.02 times that at 100 m. This is because, in the later stages of heat extraction, the working fluid flow is no longer the main factor affecting reservoir subsidence. The subsidence is closely related to the reservoir temperature. The existence of vertical fractures increases the heat exchange area between the matrix and the fractures, enhancing heat extraction rate and leading to an increase in the average subsidence of the reservoir. Since the area far from the injection well maintains high temperature, adding vertical fractures in this area most effectively increases the heat extraction rate but also results in the highest average subsidence of the reservoir. As shown in Fig 13(c), the more vertical fractures there are, the greater the average subsidence of the reservoir is. After 30 years of heat extraction, the average reservoir subsidence with five vertical fractures is approximately 1.09 times that with one vertical fracture.

**Fig 14:**
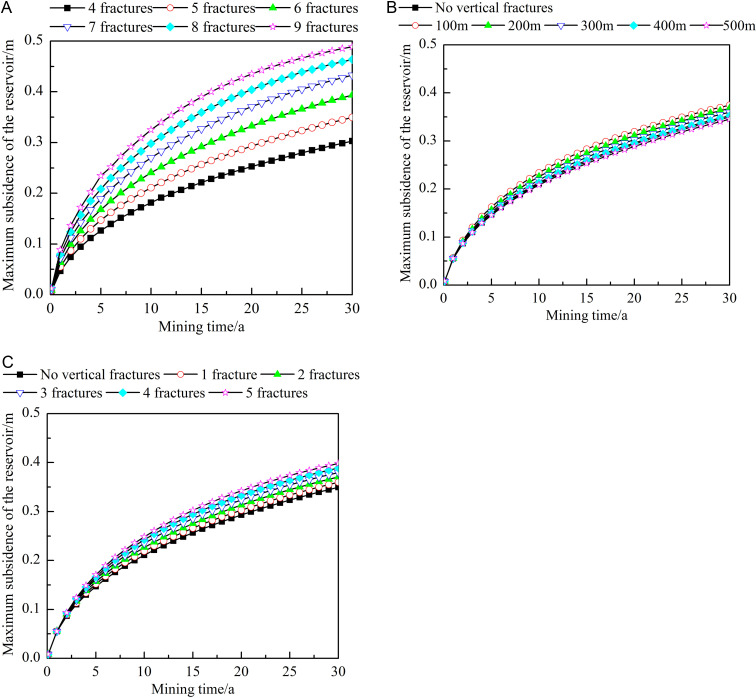
Influences of different fracture characteristics on the maximum reservoir subsidence. A) Number of horizontal fractures. B) Locations of the vertical fractures. C) Number of vertical fractures. The dots in the figure represent the maximum reservoir subsidence at different heat extraction times (0-30 year). The figure (a) shows the maximum reservoir subsidence when the number of horizontal fractures is 4-9. The figure (b) shows the maximum reservoir subsidence when no vertical fractures or the vertical fracture is 100-500m away from the injection well. The figure (c) shows the maximum reservoir subsidence when no vertical fractures or the number of vertical fractures is 1-5.

Fig 14 shows the influence of different fracture characteristics on the maximum subsidence of the reservoir. The location of the maximum reservoir subsidence is most likely to trigger changes in geological conditions. Studying this aspect can help detect and address potential geological hazards in the timely manner to ensure the safety and stability of the heat extraction process. Figs 12 and 14 show that the maximum subsidence is located at the junction between the caprock and the reservoir near the injection well. During the extraction process, the maximum subsidence of the reservoir shows a gradual increasing trend. As shown in Fig 14(a), the larger the number of horizontal fractures is, the greater the maximum subsidence of the reservoir. After 30 years of extraction, the maximum subsidence with 9 horizontal fractures is about 1.61 times that with 4 fractures. As shown in Fig 14(b), when vertical fractures are close to the injection well, they increase the maximum subsidence of the reservoir. After 30 years of heat extraction, the maximum subsidence when the vertical fractures are 500 m from the injection well is approximately 92.13% of that at 100 m. This is because the closer the vertical fractures are to the injection well, the larger the temperature difference between this area and the initial state of the reservoirs. As a result, the shrinkage and fracture of the rock in this area are the most severe, eventually forming a positive feedback mechanism that significantly increases subsidence. As shown in Fig 14(c), the larger the number of vertical fractures, the greater is the reservoir subsidence. After 30 years of heat extraction, maximum subsidence of the reservoir with five vertical fractures is approximately 1.11 times that in the case with one vertical fracture.

#### Influence of fracture characteristics on the stress field.

**Fig 15:**
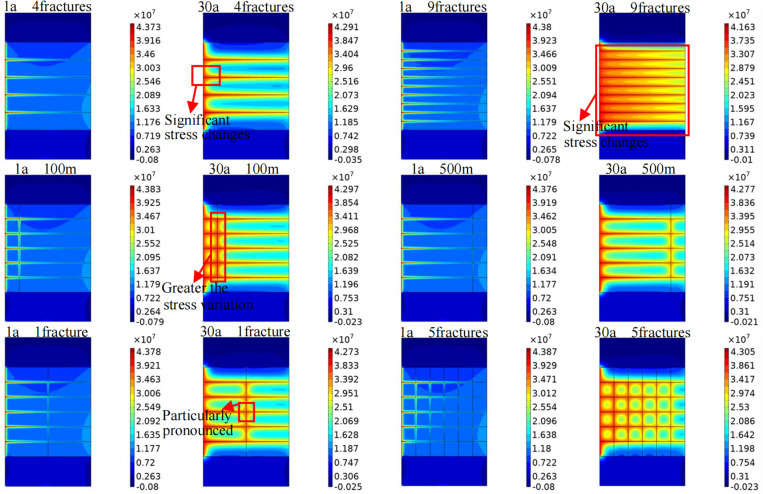
Influences of different fracture characteristics on the stress field in the target area. 1a and 30a represent different heat extraction times. The figure in the first row shows the variation pattern of the stress field in the target area when the number of horizontal fractures is 4 and 9. The figure in the second row shows the variation of stress field in the target area when the vertical fracture is 100m and 500m away from the injection well. The figure in the third row shows the variation pattern of the stress field in the target area when the number of vertical fractures is 1 and 5.

The change in the in situ stress determines the duration during which the reservoir can maintain stable extraction, and fractures constitute the main factor influencing the in situ stress of the reservoir. Fig 15 shows the influence of different fracture characteristics on the stress field in the target area. With continuous fluid injection and continuous heat exchange between water and the rock, the original stress state of the reservoir changes, and the stress field varies accordingly. During extraction, the variation range of the stress field is similar to that of the temperature field. Therefore, only the influence range and mechanism of the stress field under the different fracture characteristics at the later stage of extraction are investigated. Horizontal fractures are the main channels for seepage and heat transfer. Due to the increase in fracture connectivity, stress transfer paths are formed around fractures, causing significant stress changes in the vicinity. The closer to the horizontal fractures, the higher the stress value and the poorer the crustal stability. With increasing number of horizontal fractures, the influence range of the reservoir stress field increases. When nine horizontal fractures are implemented, almost the entire reservoir exhibits significant stress changes. The closer the vertical fractures are located to the injection well, the greater the stress variation, especially in the junction area between the horizontal and vertical fractures, where stress concentration is particularly notable. When the number of vertical fractures is increased, the influence range of the in situ stress accordingly increases significantly.

**Fig 16:**
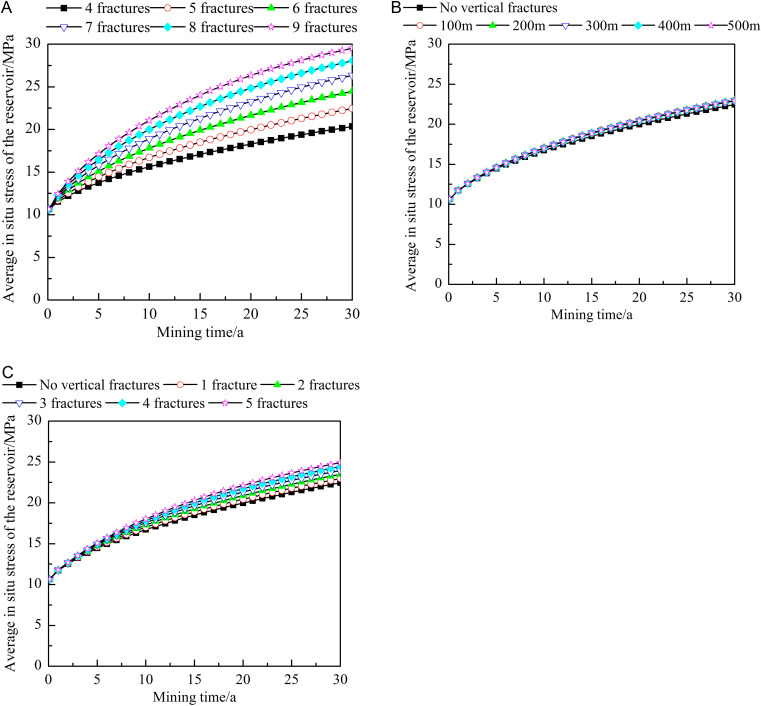
Influences of different fracture characteristics on the average in situ stress in the reservoir. A) Number of horizontal fractures. B) Locations of the vertical fractures. C) Number of vertical fractures. The dots in the figure represent the average in situ stress at different heat extraction times (0-30 year). The figure (a) shows the average in situ stress when the number of horizontal fractures is 4-9. The figure (b) shows the average in situ stress when no vertical fractures or the vertical fracture is 100-500m away from the injection well. The figure (c) shows the average in situ stress when no vertical fractures or the number of vertical fractures is 1-5.

Fig 16 shows the influence of different fracture characteristics on the average in situ stress of the reservoir. During heat extraction, the average in situ stress of the reservoirs generally shows an increasing trend, with a rapid increase at the early stages of heat extraction and a slight slowdown at the later stages of heat extraction. The characteristic changes are directly related to the continuous injection of the working fluid. As shown in Fig 16(a), the larger the number of horizontal fractures is, the greater the increase in the average in situ stress of the reservoir. After 30 years of extraction, the average stress obtained with nine horizontal fractures is approximately 1.45 times that obtained with four fractures. Therefore, although an increase in the number of horizontal fractures increases the heat extraction efficiency, it causes rapid in situ stress changes. When the stress exceeds the bearing capacity of the rock, fracture channels may be redistributed, thus affecting the final productivity. As shown in Fig 16(b), when the vertical fractures are located close to the injection well, the average in situ stress of the reservoir slightly increases, but the location of the vertical fractures is not the main factor influencing the average in situ stress. As shown in Fig 16(c), the larger the number of vertical fractures is, the higher the average in situ stress of the reservoir. After 30 years of extraction, the average in situ stress obtained with five vertical fractures is approximately 1.09 times that obtained with one vertical fracture.

**Fig 17:**
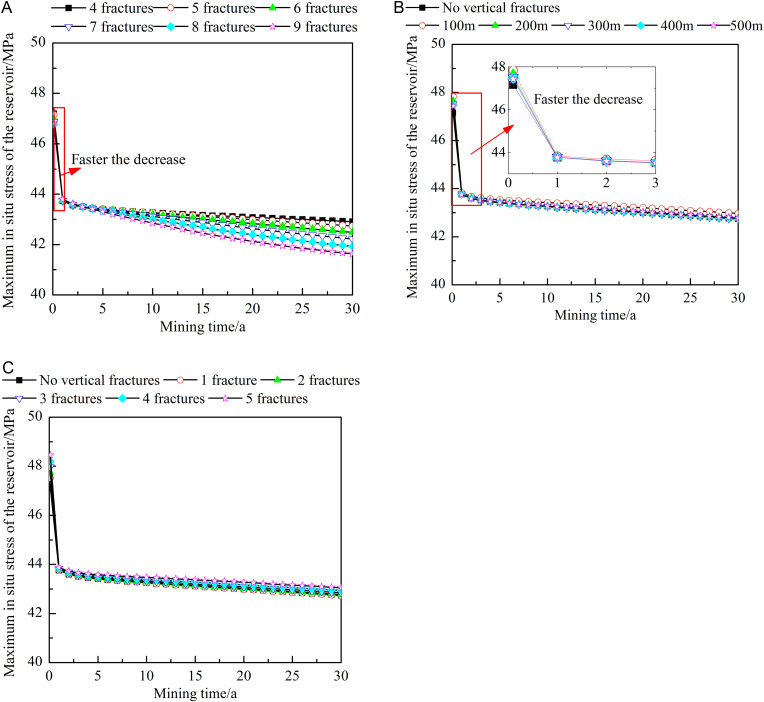
Influences of different fracture characteristics on the maximum in situ stress in the reservoir. A) Number of horizontal fractures. B) Locations of the vertical fractures. C) Number of vertical fractures. The dots in the figure represent the maximum in situ stress at different heat extraction times (0-30 year). The figure (a) shows the maximum in situ stress when the number of horizontal fractures is 4-9. The figure (b) shows the maximum in situ stress when no vertical fractures or the vertical fracture is 100-500m away from the injection well. The figure (c) shows the maximum in situ stress when no vertical fractures or the number of vertical fractures is 1-5.

Fig 17 shows the influence of different fracture characteristics on the maximum in situ stress of the reservoir. Combining Figs 15 and 17, the maximum in situ stress of the reservoir occurs at the intersection of the injection well and the horizontal fractures, which is the potential fracture surface of the rock mass. As the extraction process progresses, the variation in the maximum in situ stress follows a trend opposite that in the average in situ stress, demonstrating a general decline. This is mainly related to the significant consumption of heat within the reservoir. Notably, the maximum in situ stress of the reservoir decreases most significantly during the first year of extraction. Under the different fracture characteristics, the maximum decrease in the in situ stress exceeds 50%. As shown in Fig 17(a), the larger the number of horizontal fractures is, the faster the decrease in the maximum in situ stress. After 30 years of extraction, the maximum in situ stress obtained with nine horizontal fractures is approximately 97.02% of that obtained with four horizontal fractures. Fig 17(b) shows that the maximum in situ stress in the presence of vertical fractures in the reservoir is slightly higher than that in their absence. When the distance from the injection well is 100 m, the maximum in situ stress is the highest. This trend is most obvious at the early stages of heat extraction, but the location of the vertical fractures exerts a relatively limited influence on the maximum in situ stress. As shown in Fig 17(c), the numbers of vertical and horizontal fractures influence the maximum in situ stress completely differently. An increase in the number of vertical fractures leads to a slight increase in the maximum in situ stress.

### Impact of fracture characteristics on the economic benefits of HDR exploitation

The number of horizontal fractures, the location of the vertical fractures, and the number of vertical fractures notably influence the economic benefits of HDR extraction. These parameters not only directly affect the efficiency of heat extraction and resource utilization but also affect reservoir stability, thereby profoundly influencing the overall economic benefits of HDR extraction.

Horizontal fractures, as the key seepage and heat exchange pathways in the reservoir, constitute the main factor determining the economic benefits of HDR exploitation. Increasing the number of horizontal fractures within a reasonable range can significantly enhance fluid mobility and heat exchange capacity levels, thereby increasing the heat extraction efficiency within a certain period and enhancing the economic benefits. However, an excessive number of horizontal fractures may result in delayed heat recovery in the reservoir, compromise reservoir stability, cause dual waste of water and geothermal resources, and ultimately negatively impact economic benefits.

The location of the vertical fractures exerts a relatively limited impact on the economic benefits of HDR extraction, but this factor still requires attention. The location of the vertical fractures plays a key role in controlling the distribution of the fluid pressure and temperature in the extraction process, thereby affecting the resource utilization efficiency and the rationality of the injection–production well layout. Therefore, it is essential to accurately obtain location information of the vertical fractures to systematically optimize the layout of the injection and production wells, reduce investment costs, and increase economic benefits.

The impact of the number of vertical fractures on the economic benefits of HDR extraction is smaller than that of horizontal fractures, but this factor should not be neglected. A moderate increase in the number of vertical fractures can enlarge the contact area between the fluid and rock while also enhancing the thermal compensation effect from the upper and lower rock layers to the reservoir, increasing the heat exchange efficiency and thus contributing to the improvement in economic benefits. However, the implementation of an excessive number of vertical fractures can alter the rock mechanical properties, increase instability in the extraction process, and result in higher extraction difficulty and costs, thus adversely affecting economic benefits.

## Discussion

### Influences of vertical fracture connectivity and the characteristics of heat transfer and seepage between the upper and lower rock layers on the temperature field

The upper and lower rock layers significantly affect the thermal compensation effect, directly influencing the range and intensity of thermal compensation and indirectly impacting the heat extraction efficiency and operational lifespan of the reservoir. Notably, vertical fractures serve as the main channels for heat transfer, compensation, and fluid flow. Therefore, it is crucial to investigate the influences of the connectivity characteristics of vertical fractures and the heat transfer and seepage characteristics of the upper and lower rock layers on the reservoir temperature field.

**Fig 18:**
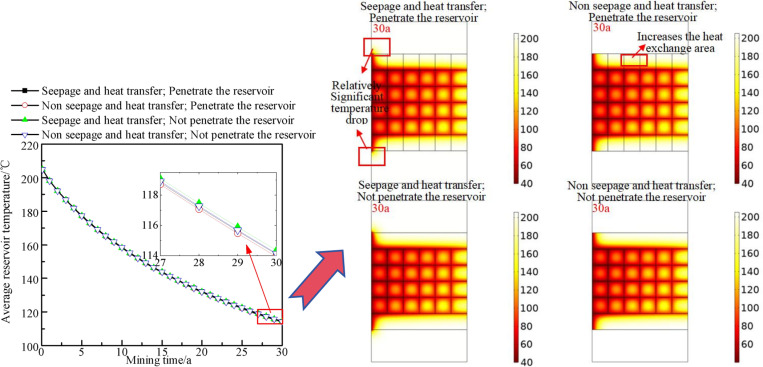
Influences of the vertical fracture connectivity and the characteristics of seepage and heat transfer between the upper and lower rock layers on the reservoir temperature field and average temperature. The dots in the left figure represent the average reservoir temperature at different heat extraction times (0-30 year). The dots of different shapes in the legend represent four different situations from top to bottom. (1) The upper and lower rock layers are seepage and heat transfer, and vertical fractures penetrate the reservoir. (2) Non seepage and heat transfer in the upper and lower rock layers, and vertical fractures penetrate the reservoir. (3) The upper and lower rock layers are seepage and heat transfer, and the vertical fractures do not penetrate the reservoir. (4) Non seepage and heat transfer in the upper and lower rock layers, and the vertical fractures do not penetrate the reservoir. The right figure shows the change law of heat extraction temperature field after 30 year under the above four conditions.

Fig 18 show the influences of the vertical fracture connectivity and the characteristics of seepage and heat transfer between the upper and lower rock layers on the reservoir temperature field and average temperature. When seepage and heat transfer between the upper and lower rock layers are considered, there is a relatively significant temperature decrease in the upper and lower rock layers near the injection well and the junction area of the reservoir, whereas other areas of the upper and lower rocks also exhibit certain temperature changes. This portion of heat flows into the reservoir to compensate for the loss occurring during heat extraction. When vertical fractures penetrate the reservoir, the temperature decrease area slightly increases. This occurs because although extending the vertical fractures accelerates the thermal compensation effect from the upper and lower rock layers to the reservoir, it also increases the heat exchange area between the matrix and fluid, with the latter imposing a greater effect than the former. To explore the influence of these two factors on the reservoir temperature field, the variation in the average reservoir temperature is investigated. At the early stages of extraction, heat is provided mainly by the reservoir area, and the connectivity of vertical fractures and the heat transfer and seepage characteristics of the upper and lower rock layers slightly influence the variation in the average reservoir temperature. As the process of extraction progresses, the effects of the characteristics of the upper and lower rock layers and vertical fractures begin to emerge. After 30 years of heat extraction, the average reservoir temperature is highest when seepage and heat transfer between the upper and lower rock layers occur and when vertical fractures do not penetrate the reservoir. When these conditions are reversed, the average temperature is lowest, with the former approximately 0.42°C higher than the latter. This further confirms the previously discussed viewpoints.

### Limitations and prospects

In this study, an EGS multifracture heat extraction model was established, and the influences of different fracture characteristics on reservoir productivity and the temperature, displacement, and stress fields were evaluated, providing a basis for evaluating productivity and predicting various reservoir fields. Nevertheless, there remain deficiencies in the process of model establishment and simulation that must be investigated further, and future research directions are proposed.

The model adopts a parallel and uniform multifracture system. The thickness of each part of the rock mass is set to be the same, and the working fluid flows uniformly toward each fracture, representing an idealized state of heat extraction. However, in the actual heat extraction process, fractures are distributed randomly and nonuniformly, with different orientations, lengths, widths, and densities. Therefore, the actual flow length and heat exchange area of the fluid between the injection and production wells are greater than those based on the results obtained in this study. Due to the complexity of the fracture network, the seepage rate and the degree of heat transfer of the fluid in the fractures also vary. The fluid flows very fast in some areas and very slow in others. Some areas exhibit heat concentration while others contain insufficient heat. Therefore, predicting the actual fracture characteristics, fluid flow rate and heat exchange effect should be addressed next.

To address this challenge, future efforts should focus on integrating methods such as data investigation, field surveys, and experimental exploration to comprehensively explore the intrinsic connections between fractures and seepage characteristics. It is expected that high-pressure water jet technology will be employed to accurately prefabricate different types of fracture channels. High-temperature cooling experiments should be conducted to simulate the real heat extraction process for assessing the influences of thermal shock on fracture development and seepage characteristics. Acoustic technology and high-resolution computed tomography (CT) scanning can be applied to reconstruct the fracture channels and obtain detailed information on the fracture geometry. Finally, an improved triaxial permeability tester can be used in HDR permeability experiments to explore the variations in the characteristic flow rate and seepage time and subsequently calculate the seepage velocity. The goal is to provide a solid foundation for the study of HDR fracture characteristics, seepage features, and heat extraction efficiency.

## Conclusions

Based on geological survey data from the Xudong fault zone in the Songliao Basin and via the use of COMSOL software, an EGS multifracture heat extraction model was established to investigate the variations in reservoir productivity and the temperature, displacement and stress fields under different numbers of horizontal fractures, locations of vertical fractures, and numbers of vertical fractures. Moreover, the mechanisms through which vertical fracture connectivity and the characteristics of heat transfer and seepage between the upper and lower rock layers influence the temperature field were elucidated. The conclusions are as follows:

(1) An increase in the number of horizontal fractures triggers multiple complex effects, which not only promote the increase of production flow and thermal compensation effect, but also help to achieve higher production temperature and the heat extraction efficiency at the early and middle stages of heat extraction. However, correspondingly, as the process of heat extraction continues, it accelerates heat loss, resulting in a significant decrease in the production temperature and the heat extraction efficiency at the later stages of heat extraction. In addition, the influence range of each field in the reservoir significantly increases, causing a notable decrease in both the average and maximum reservoir temperatures. The exacerbates reservoir subsidence and weakens the rock structure, resulting in significant increases in the average subsidence, maximum subsidence, and average in situ stress of the reservoir and a decrease in the maximum in situ stress.
(2) The influences of an increase in the number of naturally occurring vertical fractures on productivity and various fields are similar to those of horizontal fractures, but with a slightly smaller magnitude. The difference lies in that the production flow decreases at the early stages of heat extraction, whereas the maximum reservoir temperature increases at the later stages of heat extraction. When vertical fractures approach the injection wells, production temperature, and heat extraction efficiency of the reservoir at the early stages of heat extraction are relatively low. As heat extraction progresses, the trend is gradually reversed. The average subsidence remains low, with the maximum subsidence, average in situ stress, and maximum in situ stress slightly increasing.
(3) The upper and lower rock layers impose a thermal compensation effect on the reservoir, thereby compensating for the heat loss in the extraction process and extending the duration of reservoir heat extraction. When vertical fractures penetrate the reservoir, although the thermal compensation effect is accelerated, the heat exchange area also increases. However, the latter generates a greater influence than the former, resulting in a lower average reservoir temperature than when vertical fractures do not penetrate the reservoir. The influence of these two factors on the reservoir temperature field is particularly notable at the later stages of heat extraction.

In summary, in heat extraction from HDR reservoirs, we must fully recognize the enormous economic value while remaining aware of the potential environmental risks and hazards that may be triggered by this process. By weighing and balancing both factors and constructing an appropriate fracture system with planned extraction, we can effectively utilize geothermal resources without overexploitation, which can lead to irreversible ecological disasters.

## Supporting information

S1 File Influences of different fracture characteristics on the average reservoir temperature. (XLSX)
